# Pulsed-Focused Ultrasound Provides Long-Term Suppression of Epileptiform Bursts in the Kainic Acid-Induced Epilepsy Rat Model

**DOI:** 10.1007/s13311-022-01250-7

**Published:** 2022-05-17

**Authors:** Po-Chun Chu, Hsiang-Yu Yu, Cheng-Chia Lee, Robert Fisher, Hao-Li Liu

**Affiliations:** 1 Department of Electrical Engineering, National Taiwan University, Taipei, Taiwan 106; 2 Department of Neurology, Taipei Veteran General Hospital, Taipei, Taiwan; 3 School of Medicine, National Yang Ming Chiao Tung University, Taipei, Taiwan; 4 Department of Neurosurgery, Taipei Veteran General Hospital, Taipei, Taiwan; 5 Department of Neurology, Stanford Neuroscience Health Center, Stanford University School of Medicine, 213 Quarry Road, Room 4865, Palo Alto, CA 94304-5979 USA

**Keywords:** Focused ultrasound, Neuromodulation, Temporal lobe epilepsy, Kainic acid model

## Abstract

Focused ultrasound (FUS) has potential utility for modulating regional brain excitability and possibly aiding seizure control; however, the duration of any beneficial effect is unknown. This study explores the efficacy and time course of a short series of pulsed FUS in suppressing EEG epileptiform spikes/bursts in a kainic acid (KA) animal model of temporal lobe epilepsy. Forty-four male Sprague–Dawley rats were recorded for 14 weeks with EEG while software calculated EEG numbers of epileptiform spikes and bursts (≥ 3 spikes/s). Four regimens of FUS given in a single session at week 7 were evaluated, with mechanical index (MI) ranging from 0.25 to 0.75, intensity spatial peak temporal average (*I*_SPTA_) from 0.1 to 2.8 W per cm^2^, duty cycle from 1 to 30%, and three consecutive pulse trains for 5 or 10 min each. Controls included sham injections in four and KA without FUS in eleven animals. Histological analysis investigated tissue effects. All animals receiving KA evidenced EEG spikes, averaging 10,378 ± 1651 spikes per 8 h and 1255 ± 199 bursts per 8 h by weeks 6–7. The KA-only group showed a 30% of increase in spikes and bursts by week 14. Compared to the KA-only group, spike counts were reduced by about 25%, burst counts by about 33%, and burst durations by about 50% with FUS. Behavioral seizures were not analyzed, but electrographic seizures longer than 10 s declined up to 70% after some FUS regimens. Repeated-measure ANOVA showed a significant effect of higher intensity and longer sonication duration FUS treatment using 0.75-MI, *I*_SPTA_ 2.8 W/cm^2^, 30% duty cycle for 10-min sonications (group effect, *F* (4, 15) = 6.321, *p* < 0.01; interaction effect, *F* (44, 165) = 1.726, *p* < 0.01), with the hippocampal protective effect lasting to week 14, accompanied by decreased inflammation and gliosis effect. In contrast, spike and burst suppression were achieved using an FUS regimen with 0.25-MI *I*_SPTA_ 0.5 W/cm^2^, 30% duty cycle for 10-min sonications. This regimen reduced inflammation and gliosis at weeks 8–14 and protected hippocampal tissue. This study demonstrates that low-intensity pulsed ultrasound can modulate epileptiform activity for up to 7 weeks and, if replicated in the clinical setting, might be a practical treatment for epilepsy.

**Supplementary Information:**

The online version contains supplementary material available at 10.1007/s13311-022-01250-7.

## Introduction

Epilepsy is diagnosed in approximately 50 per 100,000 individuals per year [1]. Temporal lobe epilepsy (TLE) is the most common form of epilepsy in adults, characterized by focal aware (previously simple partial) seizures or focal-impaired awareness (previously called complex partial [2]) seizures and seizures that may generalize secondarily. Accompanying features include ictal and interictal electroencephalographic (EEG) abnormalities, hippocampal sclerosis, and often behavioral dysfunction [3–5].

Anti-seizure medications are the foundation of epilepsy therapy but approximately 20–40% of all epilepsy patients have drug-resistant epilepsy [6–8]. Surgical treatment may be effective in patients with refractory seizures, but not all patients are candidates, surgery may not be curative, and it can cause serious adverse events [6, 9, 10]. Brain stimulation techniques, including vagal nerve stimulation, responsive neurostimulation, and deep brain stimulation of thalamus, are palliative but also somewhat invasive [11]. Noninvasive transcranial magnetic or direct current stimulation is difficult to focus at specific target [12–15] and not approved as standard therapies. Thus, additional noninvasive therapeutic approaches are needed.

Transcranial focused ultrasound (FUS) is a novel technology that can non-invasively target deep brain tissue. Low-intensity FUS can modulate neuronal activity [16, 17], directly stimulate action potentials and synaptic transmission through non-thermal activation of sodium and potassium ion channels [18, 19], and decrease excitation of primary motor cortex in human brain [20]. FUS neuromodulation has the potential to be a viable therapy for epilepsy [14, 21–23]. The neuromodulating potential of transcranial ultrasound stimulation in epilepsy was suggested by laboratory studies showing that FUS stimulation attenuates acute epileptic signals [24, 25]. However, the long-term effects of FUS neuromodulation on seizure activity have not been established, which is relevant to the potential utility of FUS in chronic clinical practice. Hakimova and associates utilized “preventive” pulsed FUS prior to the onset of kainic acid (KA)-induced seizures in an animal model and to evaluate the seizure activity suppression capability at the chronic onset phase (days 21–35 after KA injection), but the duration of the epileptic activity suppression effect was not reported [23].

KA is a cyclic analog of L-glutamate and an agonist of ionotropic KA receptors. Although there is no experimental model that reproduces all the features of TLE, the KA models, originally described by Ben-Ari et al. [26], have been accepted as a useful representation of clinical epilepsy. Intracerebral administration of KA to the amygdala or hippocampus induces behavioral seizures and neuropathological lesions that are similar to those occurring in patients with TLE, with neuronal degeneration in the CA3 region of the dorsal hippocampus, and the model has been recognized to mimic chronic clinical syndromes [5, 27].

This study aims to explore the efficacy and time course of pulsed FUS neuromodulation in the KA model. Experience with neuromodulation by repetitive transcranial magnetic stimulation suggested that multiple treatments in 1 day can provide lasting effects [15]. In this study we therefore evaluate the enduring effect on epileptiform EEG activity when conducting multiple-FUS treatments within 1 day. We additionally correlate the potential therapeutic effects with histologic examinations in order to evaluate safety of the selected ultrasound parameters.

## Methods

### Animals

All animal experiments were approved by the Institutional Animal Care and Use Committee of National Taiwan University, Taiwan (IACUC No. NTU-109-EL-00101). Animals were housed with a 12-h light/dark cycle and ad libitum access to food and water. Forty-three male Sprague–Dawley rats (290–330 g, BioLASCO Co., Ltd, Taiwan) were used. Among them, 39 received KA injections; 36 survived, 4 received non-KA sham injection, and 40 were evaluated experimentally with EEG. Of these, 25 KA-injected rats were treated with FUS and 15 rats (11 KA-injected rats and 4 non-KA-injected rats) were used as non-FUS controls. An additional four rats were used for histology analysis to examine potential brain damage after FUS sonication.

### Kainic-Acid Animal Model and Electrode Implantation

**Fig. 1:**
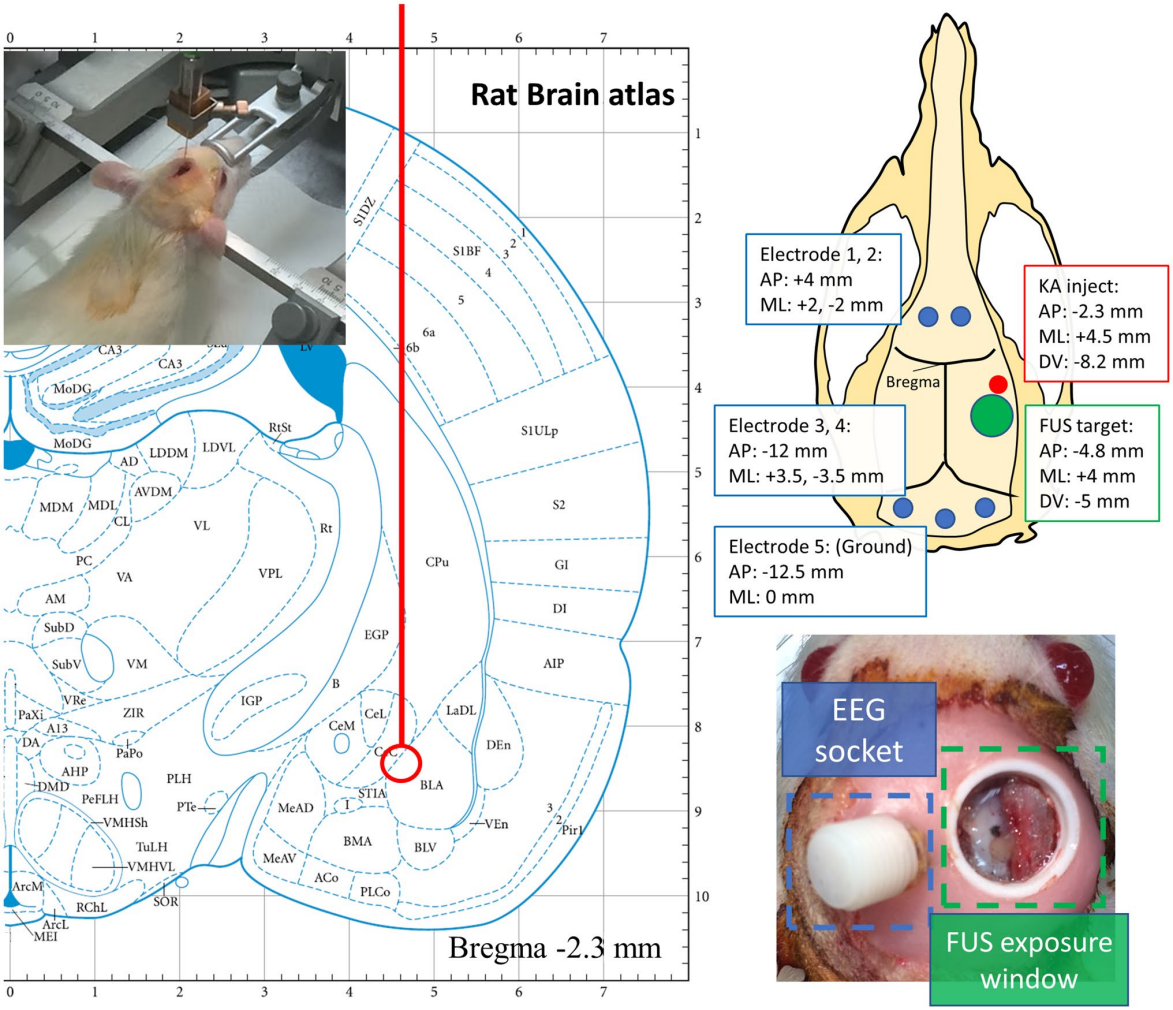
Atlas showing the KA injection site (amygdala, marked as BLA [28]), electrode position, and appearance of model

All animals were anesthetized with 2% isoflurane mixed with oxygen at 6 L/ min. The KA model was developed based on previous literature [27]. In brief, skull was exposed and opened with a dental drill. KA 750 ng was injected into the right amygdala (AP: −2.3 mm, ML: +4.5 mm, DV: −8.2 mm from bregma) through a glass capillary tubing (Polymicro Technologies, Arizona, USA) under 20 ng/s of flow rate to induce excitotoxic damage in hippocampus (Fig. 1). After injection, the glass cannula remained in place for 5 min to prevent leakage along the injecting tract. Animals without kainic acid injection were designated non-KA sham control animals.

Five stainless steel screw electrodes (1.6-mm-diameter pole; Plastics One Inc., Roanoke, Virginia, USA) were bilaterally inserted above the cerebellum (Fig. 1), at AP: +4 mm, ML: ±2 mm and at AP: −12 mm, ML: ±3.5 mm. A ground electrode was placed at AP: −12.5 mm, ML: 0 mm. All electrodes were connected to an EEG socket. A small polylactic acid window was placed for locating FUS exposure above the right hippocampus (AP: −4.8 mm, ML: +4 mm from bregma) (Fig. 1). Dental acrylic secured the electrodes, EEG socket, and the FUS exposure window.

### FUS Setup and Parameter Design

**Fig. 2:**
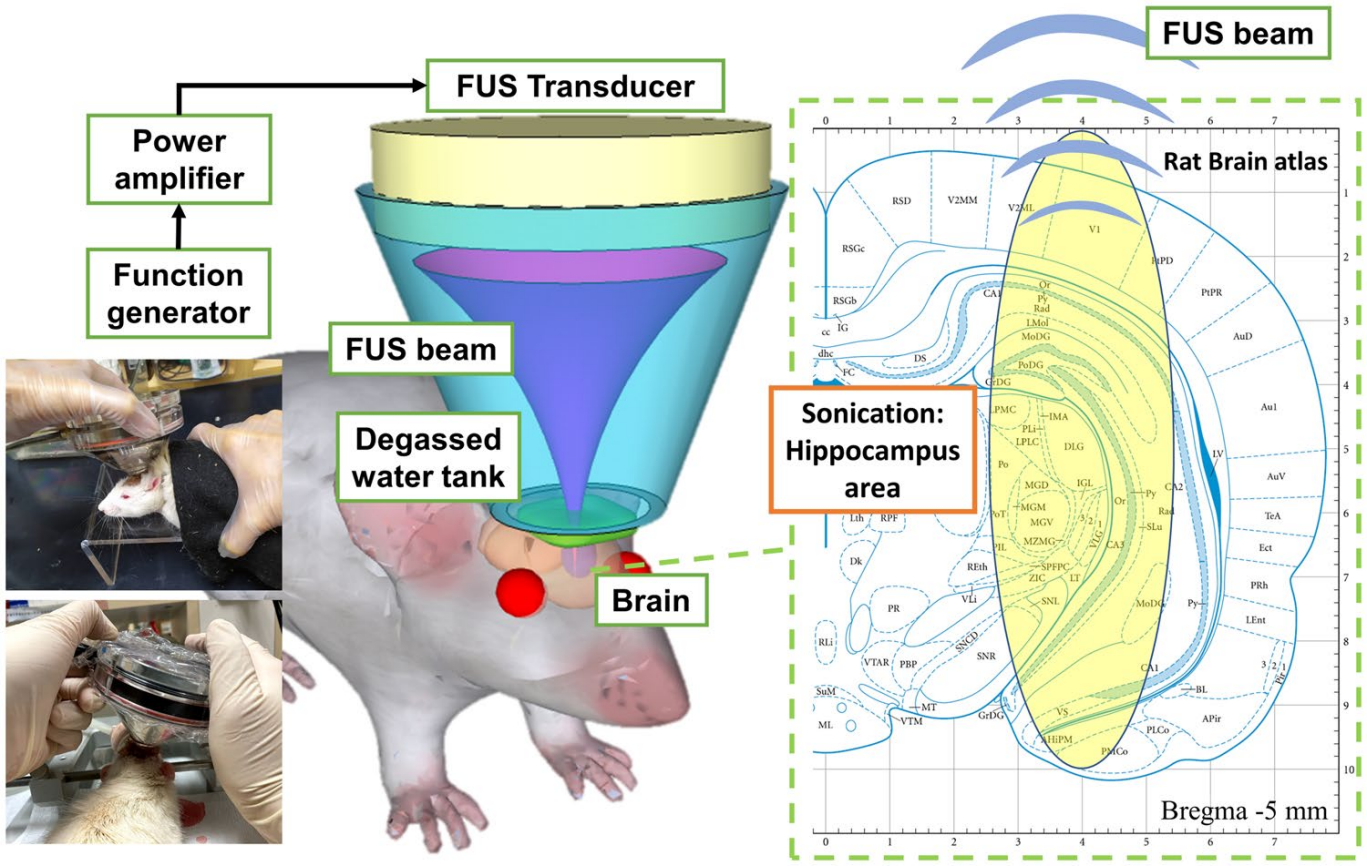
Conceptual schematic of the sonication setup and topographical sonication depth, which targeted at hippocampus area (marked as CA1, CA2, and CA3 in the atlas [28])

A focused ultrasound transducer (SonicConcept, USA; fundamental frequency = 0.5 MHz, radius curvature = 64.3 mm) delivered pulsed signals of 0.5 MHz guided by a function generator (A33420, Agilent, USA) and amplified by a radiofrequency power amplifier (240L, E&I, USA). The acoustic pressure was measured by a needle-type hydrophone (Reson, TC 4038, Goleta, California, USA) in a free field filled with deionized/degassed water. The diameter and length of the − 6 dB dimension of pressure field were 2 mm and 12 mm, respectively, and was aimed at the right hippocampus (Fig. 2).

Based on our previous investigation [25], FUS parameters were chosen as listed in Table 1. FUS exposure level was set at 0.75 MI (mechanical index) in groups 3, 4, and 5 and 0.25 MI in group 6. The duty cycle ranged from 1 to 30% (groups 3, 4, and 5), which produced FUS exposure intensities (spatial-peak temporal average intensity, *I*_SPTA_ from 0.1 to 2.8 W/cm^2^. The exposure duration was either three consecutive 5-min (groups 4 and 5) or 10-min (groups 3 and 6) “on” periods, with 5-min “off” period between each “on” period. During each sonication, ultrasonic gel was used to coat the FUS exposure window and to connect to the degassed water tank (Fig. 2).

**Table 1:** Summary of FUS parameters used in individual experimental groups

**Group**	**Name**	**Mechanical index**	***I***_**SPTA**_** (W/cm**^**2**^**)**	**Duty cycle**	**Duration**	**Animal number (*n*)**
1	Non-KA	–	–	–	–	4
2	KA only	–	–	–	–	11
3	KA + FUS	0.75 MI	2.8	30%	10 min*3	9
4	KA + FUS	0.75 MI	0.5	5%	5 min*3	5
5	KA + FUS	0.75 MI	0.1	1%	5 min*3	6
6	KA + FUS	0.25 MI	0.5	30%	10 min*3	5

*MI* mechanical index, *I*_*SPTA*_ spatial-peak temporal average intensity. Duty cycle = percentage of time on divided by total time of a stimulus train

### FUS Sonication Protocol

**Fig. 3:**
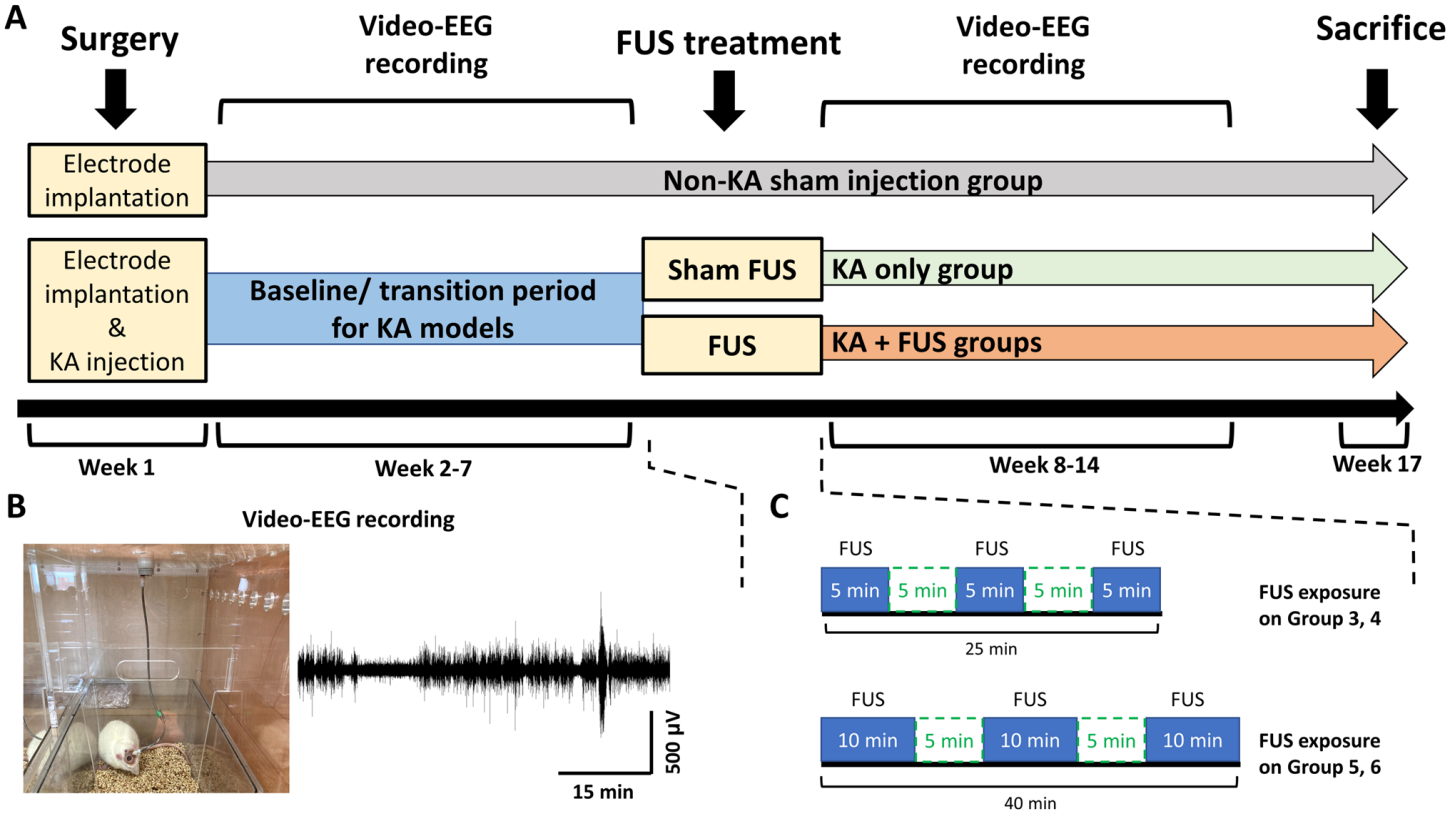
**A** Time course of experiment protocol. **B** Photos of EEG recording and EEG example. **C** Conceptual schematic of the sonication on groups 3, 4, 5, and 6

Figure 3A illustrates the FUS sonication protocol and time course. In week 1, intra-amygdala KA injection and implantation of EEG electrodes were conducted. Each EEG recording lasted for 8 h during the light period (once per week) [29], based on previous preclinical [30] and clinical [31] suggestions for long-term rodent EEG monitoring. During weeks 2–7, EEG was recorded to serve as the baseline (Fig. 3B) and to monitor the onset of EEG epileptiform bursts. During week 7, FUS was delivered while the animals were under mild anesthesia (2% isoflurane) (Fig. 3C). In 4 different treatment groups, MI ranged from 0.25 to 0.75, intensity spatial peak temporal average (*I*_SPTA_) from 0.1 to 2.8 W per cm^2^, duty cycle from 1 to 30%, and three consecutive pulse trains each were timed either for 5 or 10 min. EEG recording continued weeks 8–14 to follow the post-FUS treatment effect. The overall procedures and group characteristics are summarized in Table 1.

During FUS exposure, isoflurane was used to mildly anesthetize and stabilize the animals during sonication. Two control groups were studied: group 1 (*n* = 4) with sham injections and group 2 (*n* = 11) with KA injections but no sonications. To exclude any effects possibly caused by anesthesia, the KA-only control group (group 2) and the non-KA sham injection group (group 1) also were exposed to isoflurane inhalation in a procedure similar to that used for other FUS exposure groups (groups 3, 4, 5, and 6).

### EEG Signal Recording and Analysis

**Fig. 4:**
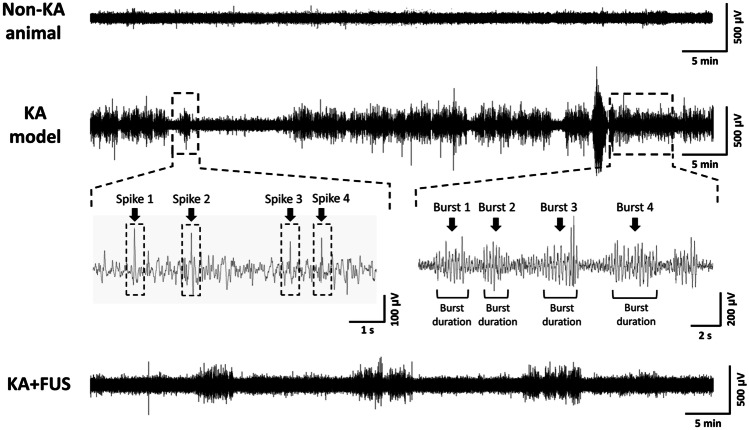
Typical example of EEG signal of non-KA model, KA model, and post-FUS KA model. Example showing examples of spikes, burst count, and burst duration

EEG signals were acquired through an EEG socket with four cortical stainless steel screw electrodes as well as a ground electrode contact positioned over the skull (Fig. 1). The EEG signal was amplified (gain = 60 dB), digitized with 500 Hz sampling (MP36, BIOPAC System, California, USA), and band-pass filtered at 4–80 Hz via an infinite impulse response filter (IIR) with a 60-Hz notch filter. Obvious artifacts, such as electromyogram greater than 0.5 mV, were truncated to the average baseline amplitude in order to minimize false spike detections. Figure 4 shows a typical EEG example.

Epileptiform spikes were detected and counted by AcqKnowledge 4.2 (BIOPAC System, California, USA). A spike was defined as a sharp deflection of amplitude at least 3 times higher than the background activity and with duration of up to 100 ms from 20 ms before the spike maximum [25, 32]. A burst was defined as three or more repetitive spikes within 1 s. Burst duration was calculated from the start of the first spike to the end of the last one (Fig. 4) [32]. For the analysis of the epileptic bursts, a blinded rater marked repetitive epileptiform spikes as well as spike bursts. Figure 4 shows representative examples of bursts. Bursts were captured and presented via EEG and spikes were also detected and counted (Fig. 4). Longitudinal weekly based EEG recordings were conducted and off-line analyzed to observe chronic epileptic signal onset. This study did not track behavioral seizures, but electrographic (EEG) seizures with consistent, repetitive spikes that persisted for more than 10 s (Fig. S1A [33]) were analyzed for some of the FUS regimens.

### Histological and Immunohistochemistry (IHC) Examinations

At time of sacrifice (week 17), non-KA sham, KA only, and KA-FUS-treated animals were anesthetized with isoflurane. Animals were sacrificed with anesthesia followed by transcardial perfusion with 0.9% saline. Brains were removed and fixed in 10% buffered neutral formalin. After fixation, the brain was cut into a series of coronal blocks and embedded in paraffin. The blocks were serially sectioned at 6 μm and stained with hematoxylin and eosin (HE) and glial fibrillary acidic protein (GFAP) with rabbit GFAP polyclonal antibody (1:100, 16,825–1-AP, Proteintech, USA). To evaluate the effect of our different FUS parameters on tissue integrity, histology was examined from rats that had not been injected with KA but were exposed to FUS at an *I*_SPTA_ of 2.8 W/cm^2^ or 0.5 W/cm^2^. The relative changes of hippocampal volume and GFAP positive signals triggered by FUS sonication were quantified while using the contralateral untreated site as the baseline (via Image J [25]).

### Statistical Analysis

Statistical analysis was performed using R Statistical Software (v. 3.6.3) and RStudio (v. 1.2.5042). EEG data were analyzed using repeated-measures ANOVA with least significant difference post hoc analysis at a criterion *p*-value of 0.05. Data are presented as mean ± standard deviation. Two-tailed/paired Student’s t-tests at criteria 0.05 were employed for histology analysis, with comparison of the target group (groups 2, 3, and 6) to the non-KA sham group.

## Results

### Longitudinal EEG Analysis

**Fig. 5:**
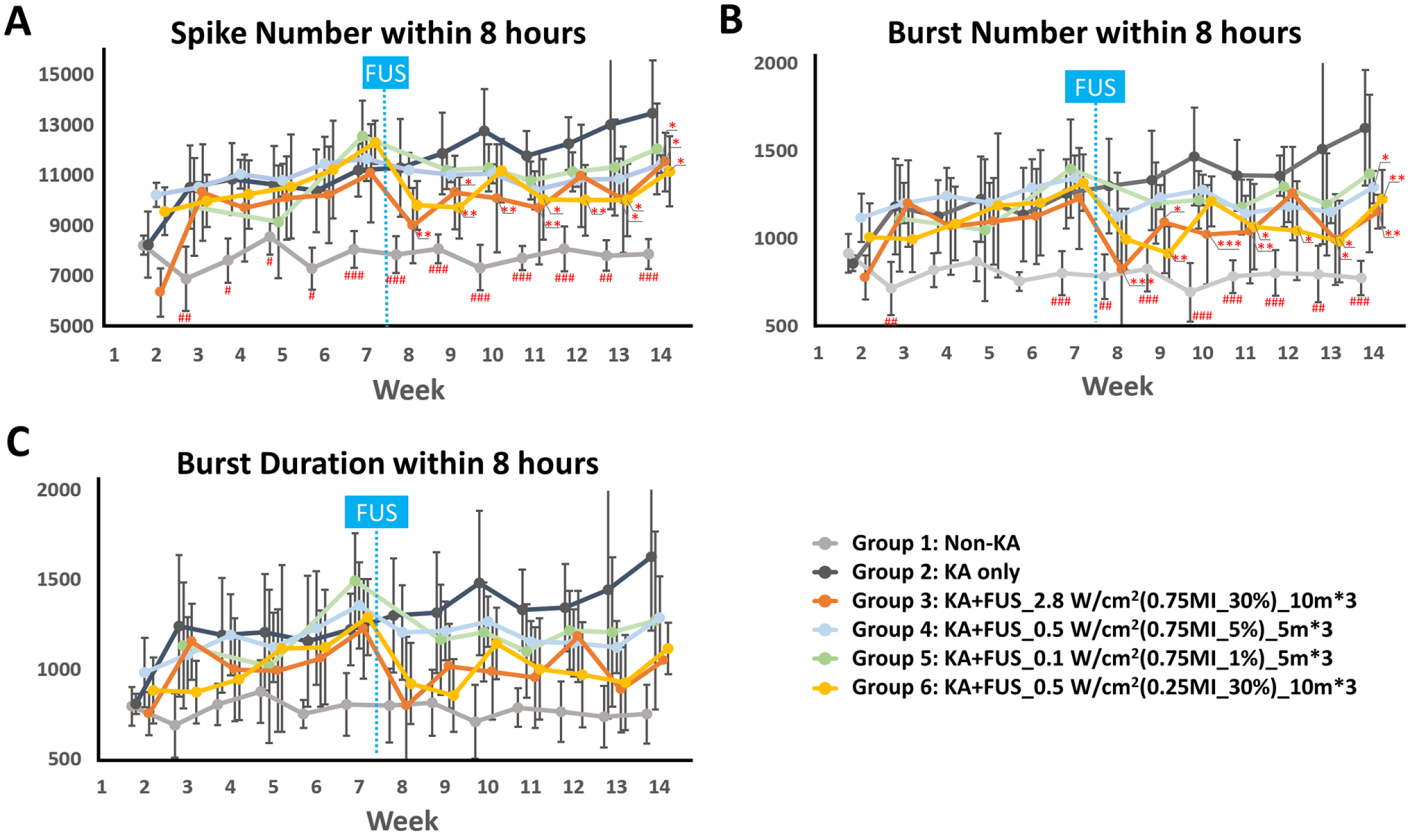
Comparison of the EEG signals for various groups (observation period: week 2–14, within 8 h per week). **A** Spike number. **B** Burst number. **C** Burst duration. FUS sonications were delivered at the end of week 7 (marked as blue dashed line). ^#^ denotes significant difference between the non-KA group and KA only group, *p* < 0.05; ^##^*p* < 0.01; ^###^*p* < 0.001. *denotes significant difference between KA + FUS group and KA only group, *p* < 0.05; **p < 0.01; ***p < 0.001

After KA intra-amygdala injection, convulsive status epilepticus developed lasted at least 4 h in the Sprague–Dawley rats. Thirty-six of the thirty-nine Sprague–Dawley rats (92%) survived after the kainic acid–induced status epilepticus. All KA-injected animals exhibited increasing EEG spikes and burst over time. Spikes in the group receiving KA but no FUS increased from 8241 ± 1318 (week 2) to 10,378 ± 1651 (week 6), which was significantly different from animals with sham injections (group 1) (*p* < 0.05) (Fig. 5A). After week 6, the animals receiving KA had bursts per 8 h increasing from 859 ± 45 (week 2) to 1255 ± 199 (week 7, Fig. 5B), again significantly different from the non-KA group (group 1) (*p* < 0.05). Spike and burst numbers in FUS-treated groups (groups 3–6, which also were administrated KA) also were increased from 9782 ± 310 (week 2) to 11,657 ± 707 (week 7) and 1015 ± 80 (week 2) to 1276 ± 111 (week 7), respectively, and significant differences were shown at week 7 in all KA-induced group. Hence, the ultrasound treatment in groups 3–6 were conducted at the end of week 7.

Over the post-treatment follow-up period (weeks 8–14), the KA-only group exhibited a progressive increase in spike counts from 11,288 ± 1947 (week 8) to 13,457 ± 2123 (week 14), burst counts from 1300 ± 269 (week 8) to 1631 ± 330 (week 14), and burst durations from 1300 ± 317 s (week 8) to 1626 ± 409 s (week 14), respectively. Repeated-measures ANOVA indicated that the magnitude of the decrease of spiking differed among the groups within weeks (group effect, *F* (4, 15) = 8.97, *p* < 0.001; interaction effect, *F* (44, 165) = 1.785, *p* < 0.01; Fig. 5A). The difference in spike number changes among the groups was observed 1 week after sonication (week 8) (*F* (4, 30) = 6.066, *p* < 0.01) and lasted to week 14 (all *p* < 0.05).

Post hoc analysis at weeks 8–11 showed that three consecutive 10-min, 0.75 MI, *I*_SPTA_ 2.8 W/cm^2^ sonications (group 3) induced the most significant suppressive effect (up to 20.3% spike reduction when comparing with KA-only group; *p* < 0.01) (Fig. 5A). The spike reduction effect seemed to gradually diminish after about 4 weeks (at week 12, *p* = 0.077) but the spike count was still significantly lower (*p* < 0.05) than the KA-only control group in all of the following weeks except for week 12.

To minimize exposure intensity for maximum safety, the original duty cycle of 30% was reduced to 5% and in other experiments to 1% with a fixed pressure level of 0.75 MI, corresponding to *I*_SPTA_ of 0.5 and 0.1 W/cm^2^, respectively. The sonication time also was reduced from 10 min for each of the three treatments to 5 min (groups 4 and 5). Unfortunately, reducing the exposure energy to these levels eliminated the spike inhibition. Group 6 was formulated with an intermediate exposure with *I*_SPTA_ level of 0.5 W/cm^2^, 0.25 MI, duty cycle 30%, and 10 s pulse trains. These parameters resulted a significant reduction of spiking up to 18.2%. Effect duration for group 6 was however briefer than for the group with higher intensities, with reduced spiking for only about 2 weeks, returning to baseline levels of spiking at week 10. Spike counts were still significantly lower than counts for corresponding KA-only controls at weeks 11–14.

Burst counts (Fig. 5B) and durations (Fig. 5C) showed similar time courses to those of spike counts (Fig. 5A). Repeated-measures ANOVA revealed a significant effect of being in the FUS group (*F* (4, 15) = 6.321, *p* < 0.01; interaction effect, *F* (44, 165) = 1.726, *p* < 0.01). Reduction of burst counts differed by week 8 (*F* (4, 30) = 4.727, *p* < 0.01) and endured during all weeks 9–14 (all *p* < 0.05).

Post hoc analysis at weeks 8–11 showed that burst counts in the *I*_SPTA_ 2.8 W/cm^2^ exposure (group 3) were 36.7% less than those for the KA-only group at week 8 (*p* < 0.001) and the effect lasted for 4 weeks (*p* < 0.05, Fig. 5B). The trend of decreasing burst numbers was most pronounced at week 12 but decreased again at weeks 13 and 14 (*p* < 0.05). Burst counts were significantly lower than those for the KA-only control group in all weeks after sonication, except for week 12 (all *p* < 0.05, except week 12). FUS with *I*_SPTA_ 0.5 W/cm^2^, 0.25-MI FUS (group 6) reduced bursts by 31.2% with an effect enduring for 2 weeks. The burst count of group 6 returned to baseline levels at week 10 but decreased again at weeks 11–14.

Burst duration (Fig. 5C) was reduced in group 3 and group 6 in parallel to burst counts (Fig. 5B), trending to a 39% reduction at week 8. The reduction did not reach significance (group effect, *F* (4, 13) = 1.75, *p* = 0.199; interaction effect, *F* (44, 165) = 1.397, *p* = 0.07).

**Fig. 6:**
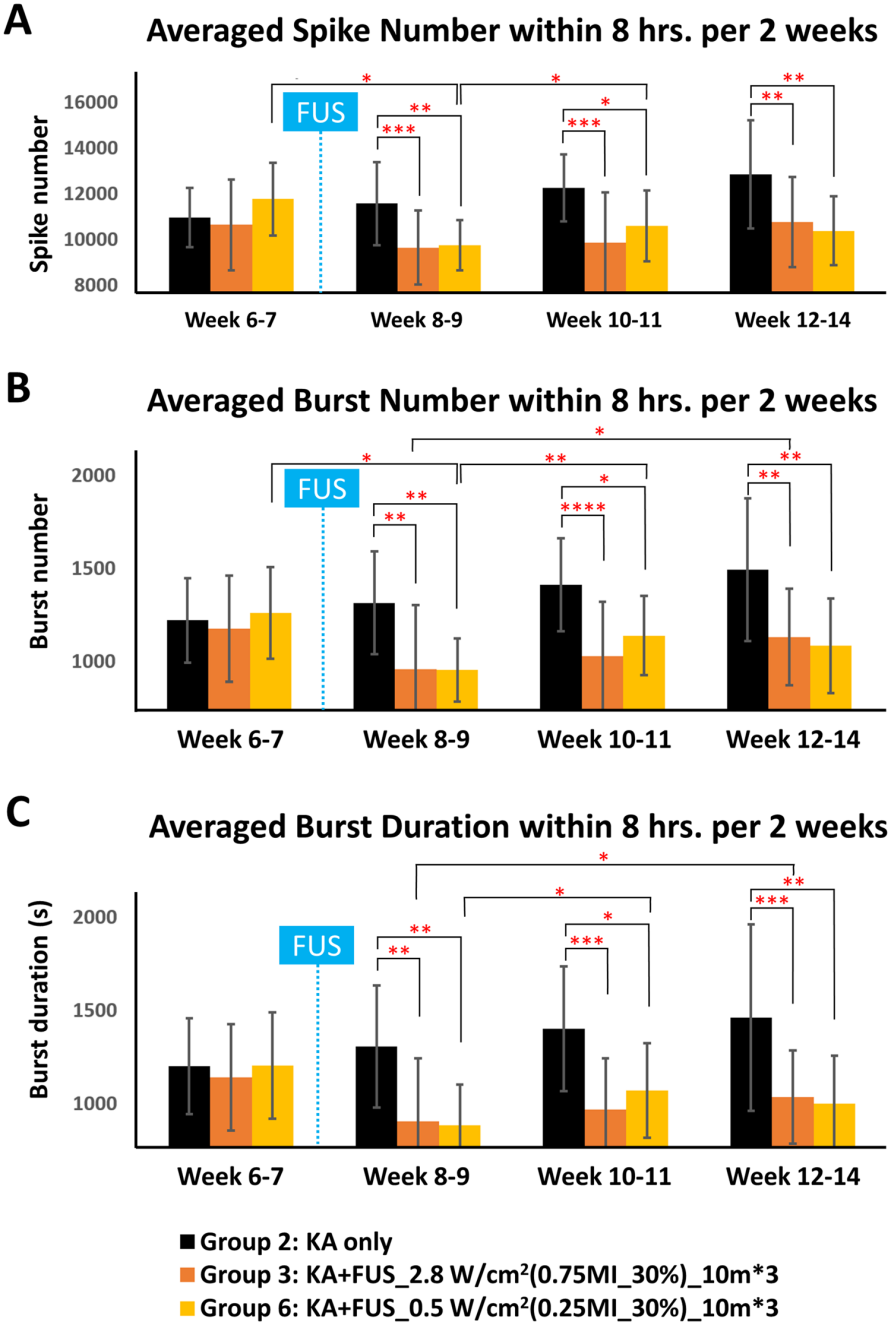
Comparison of the averaged EEG signals in various groups (observation period: week 6–14, within 8 h per 2 weeks). **A** Spike number. **B** Burst number. **C** Burst duration. FUS sonications were delivered at the end of week 7 (marked as blue dashed line). * denotes significant difference between groups, *p* < 0.05; **p < 0.01; ***p < 0.001

Re-analysis using 2-week groupings demonstrated a significant effect of FUS on burst duration (*F* (5, 32) = 13.081, *p* < 0.001; interaction effect, *F* (11.55, 73.95) = 2.745, *p* < 0.01, Fig. 6A); burst number: group effect, *F* (5, 32) = 10.795, *p* < 0.001; interaction effect, *F* (15, 96) = 2.594, *p* < 0.01 (Fig. 6B); burst duration: group effect, *F* (5, 32) = 8.126, *p* < 0.001; interaction effect, *F* (15, 96) = 2.506, *p* < 0.01 (Fig. 6C). Effects with this analysis endured to week 14 (spike number: *F* (5, 35) = 8.887, *p* < 0.001; burst number: *F* (5, 35) = 5.456, *p* < 0.001; burst duration: *F* (5, 35) = 4.669, *p* < 0.01).

In this bi-week reanalysis, the post hoc analysis showed that reduction of spike number, burst number, and burst duration reduction when compared to the KA-only group was 16.6%, 27.2%, and 30.7%, respectively, with 0.75 MI, 2.8 W/cm^2^ exposure (group 3), lasting for weeks 8–14 (Fig. 6). Spiking started to recover at weeks 12–14, but 2.8 W/cm^2^ FUS sonication seemed to successfully suppress the epileptic signals for whole observation period (weeks 8–14). A low-intensity group using 0.25 MI, 30% duty cycle, *I*_SPTA_ 0.5 W/cm^2^ (group 6) produced a reduction of epileptic signals at weeks 8–9 (spike number 15.8% lower, burst number 27.4% lower, and burst duration 32.2% lower). Spikes and bursts slightly increased at weeks 10–11 but decreased at the following weeks 12–14 (*p* < 0.05). Spike number, burst number, and burst duration were significantly lower than the numbers for the KA-only group at weeks 8–14.

Electrographic seizure analysis of EEG events with sustained spiking for at least 10 s was conducted for groups 2, 3, and 6, with 5 subjects selected randomly per group. The KA-only group exhibited a progressive increase in seizure frequency from 5.1 ± 1.6 (weeks 2–7) to 6.8 ± 2.37 seizures per week (weeks 8–14). Animals receiving KA and sham FUS showed a non-significant 106.7 ± 136.1% increase of the electrographic seizure frequency (Fig. S2B). In contrast, electrographic seizure frequencies declined from 5.4 ± 2.71 (weeks 2–7) to 4.3 ± 0.4 seizures per week (weeks 8–14) after 0.75 MI, 2.8 W/cm^2^ and from 4.2 ± 0.98 (weeks 2–7) to 1.8 ± 1.2 seizures per week (weeks 8–14) after 0.25 MI, 0.5 W/cm^2^ sonications (Fig. S2B). In contrast, the electrographic seizure frequencies were reduced by –34.8 ± 22.5% after treatment with 0.75 MI, 2.8 W/cm^2^ sonication (group 3) and reduced by –70 ± 60% after 0.25 MI, 0.5 W/cm^2^ sonication (group 6), respectively, where the later one presented significant difference when compared to group 2 (*p* < 0.05; see Fig. S2C).

### Histological Examinations

**Fig. 7:**
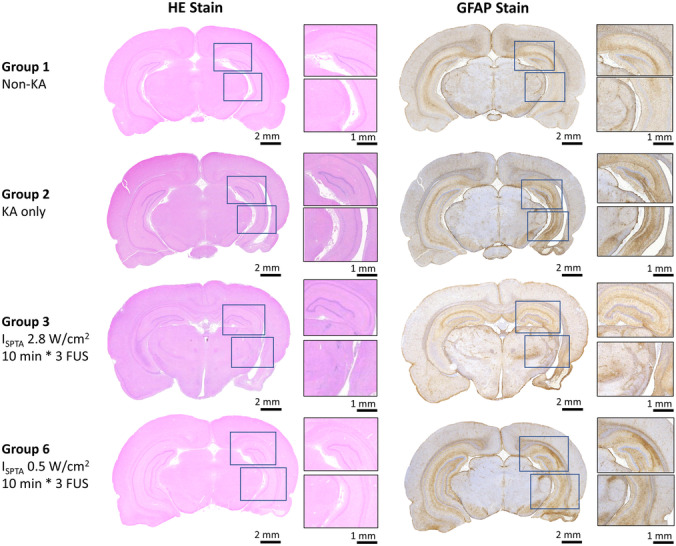
Comparison of the HE and GFAP staining among the testing group including groups 1, 2, 3, and 6

To evaluate any histological changes in the two effective groups (group 3, 0.75 MI, 2.8 W/cm^2^, and group 6, 0.25 MI, 0.5 W/cm^2^), HE and GFAP staining were conducted, as well as using non-KA (group 1)/KA-only (group 2) animals as comparison (see Fig. 7). Apparent structural changes could be observed in KA-only models; the hippocampus area shrank significantly after intracranial injection of KA, demonstrating the toxic effect of KA injection on the right (KA-injection site) hippocampus. Other structural changes of chronic KA-induced lesions included edema, hemorrhages, and partial or subtotal tissue necrosis in the right brain at the KA-injection site (observed from HE stains). GFAP positive signals were obviously increased at KA-inject hippocampus in KA-only group, which represented inflammatory response after KA injection (Fig. 7).

**Fig. 8:**
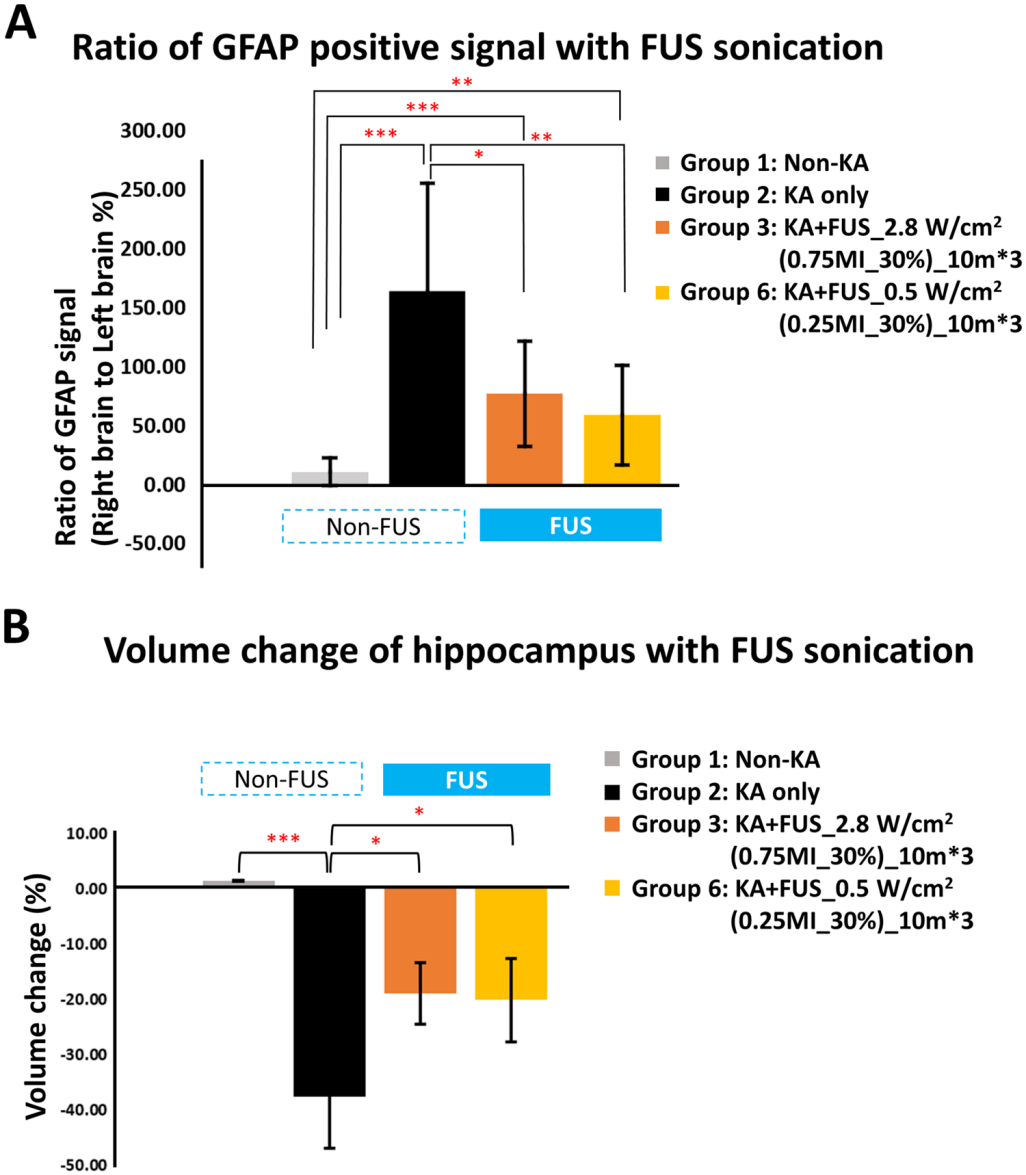
Comparison of the hippocampus volume and GFAP positive signal among the testing group, including groups 1, 2, 3, and 6. **A** GFAP-positive signal change, with the contralateral hippocampus as the basis. **B** hippocampal volume change, with the contralateral hippocampus as the basis. * denotes a significant difference between groups, *p* < 0.05; **p < 0.01; ***p < 0.001

In group 3, with 0.75-MI sonication, animals showed various degrees of inflammatory cell infiltration and one showed tissue necrosis in FUS-treated hippocampus/thalamus, with the degree comparable to that of the KA-only animals (group 2; see Fig. 7). The same ultrasound parameters (0.75 MI, 2.8 W/cm^2^, 30%, three 10-min pulse trains) induced inflammatory-like gliosis in one normal animal not receiving KA (supplementary Fig. S2, *n* = 2). In the KA-only group (group 2), GFAP markers were increased by 164.3 ± 91.7% (*p* < 0.001 when compared to the non-KA sham injection group (11.4 ± 11.6%); see Fig. 8A). Significantly less gliosis appeared after FUS treatment with 0.75 MI, *I*_SPTA_ 2.8 W/cm^2^ (group 3; 77.5 ± 44.6%; *p* < 0.05 when compared with the KA-only group; see Fig. 8A).

FUS could reduce tissue damage, such that three consecutive 10-min 0.25-MI, 0.5 W/cm^2^ FUS treatments in group 6 significantly normalized the inflammatory-like gliosis at the right hippocampus site closest to delivery of FUS, with additional improvements in thalamic inflammation and tissue necrosis. These parameters produced neither gliosis nor necrosis in normal animals (supplementary Fig. S2, *n* = 2). In fact, the GFAP-positive signal in group 6 was significantly reduced when comparing to KA-only animals (55.4 ± 42%; *p* < 0.01; see Fig. 8A).

The hippocampal volume changes among groups 1, 2, 3, and 6 were analyzed quantitatively and are presented in Fig. 8B. Animals of the non-KA group 1 presented a symmetric hippocampal volume between hemispheres, whereas the hippocampal volumes in the KA-injected hemisphere were significantly smaller, indicating severe KA-induced hippocampal damage (–37.7 ± 9.2% compared to the contralateral hippocampus; *p* < 0.001). With FUS sonication, volume changes in KA-injection hippocampus significantly reduced to –18.9 ± 5.6% and –20.2 ± 7.5% in group 3 and group 6, respectively, and were observed to be significantly different from the volume changes in the KA-only group (both *p* < 0.05, respectively). No hippocampal volume restoration was observed when comparing groups 3 and 6, indicating a treatment equivalence between these two groups.

## Discussion

An FUS neuromodulation effect has been recognized for decades. Fry et al. in their pioneering work showed that visual-evoked potentials in cats returned by 30 min after ultrasound [34]. Kim et al. suppressed visual-evoked potentials in rats only during sonication, with immediate return to baseline [35]. In hippocampal slices, Rinaldi et al. showed recovery of local field potentials 25 min after sonication [36]. Previous rodent studies of modulatory FUS for pentylenetetrazol-induced [21, 25] and kainic acid-induced seizures demonstrated efficacy [23, 24, 37, 38], but did not comment on the duration of the effect. Most previous studies [21, 23–25, 37, 39] have evaluated acute seizure [25] or spike [23] reduction. To our knowledge, demonstration of FUS effects against seizures over weeks has not previously been accomplished. We observed that three consecutive 10-min FUS sonications reduced spikes and bursts for up to 7 weeks (Fig. 6), which is a time frame relevant to clinical practice [27, 40].

In this study, three consecutive 10-min sonications at intensity 0.75 MI, 2.8 W/cm^2^ induced inflammatory-like gliosis effects but reduced EEG spikes and bursts for up to 4 weeks. In contrast, epileptiform EEG burst suppression was produced for up to 2 weeks by applying three consecutive 10-min treatments with intensity of 0.25 MI, 0.5 W/cm^2^. At this intensity, no histological changes were detected by hematoxylin and eosin staining. A number of studies previously have reported that FUS intensity (i.e., MI or *I*_SPTA_) is an important factor for neuromodulation [18, 41] and similar results were also shown in our previous work [25]. In the current study, duty cycle and exposure duration played critical roles in both efficacy and injury. When MI was fixed at 0.75 MI, a 5% duty cycle with *I*_SPTA_ 0.5 W/cm^2^ suppressed epileptiform spikes and bursts better than did a 1% duty cycle (*I*_SPTA_ 0.1 W/cm^2^) by week 14. Our previous study with a pentylenetetrazol epilepsy model similarly showed that increasing the duty cycle from 8 to 30% reduced spikes [25]. In the current study, we observed that three 10-min sonications reduced epileptiform activity more effectively than did three 5-min sonications. Although spike counts rebounded to increases at weeks 10 and 12 for group 6 and group 3, respectively, 10-min sonications significantly suppressed epileptiform discharges for the entire 8- to 14-week period of observation. This observation also corresponded to our previous pentylenetetrazol model study documenting enhanced spike suppression when treatment duration was raised from 100 to 600 s [25].

Sonication with 2.8 W/cm^2^ for three 10-min pulse trains was maximally effective among our four parameter sets, but it produced tissue inflammation and gliosis. Animals not exposed to KA also showed tissue changes when exposed to *I*_SPTA_ 2.8 W/cm^2^, identifying the FUS exposure as the likely source of inflammatory gliosis. One possible cause of inflammation is bone heating transferred to brain [42]. Another possible cause of injury is intracranial reflection of standing waves [43]. FUS provides neuroprotective effects in the KA-epilepsy model [38]. FUS sonication with some parameters could reduce inflammatory GFAP markers in hippocampus and minimize the hippocampal toxic effect produced by KA. Occasional tissue necrosis and inflammation were seen in 0.75 MI, 30% duty cycle, *I*_SPTA_ 2.8 W/cm^2^ sonication (group 3). We were able to identify parameters producing no detectable tissue injury (0.25 MI, 30% duty cycle, *I*_SPTA_ 0.5 W/cm^2^; group 6), but at a cost of reducing the duration of EEG spike inhibition from at least 4 to 2 weeks [41].

Our study is subject to several limitations. Success with animal models does not always translate to clinical benefits. This is especially true for FUS, which is dependent upon penetrating skulls that are much thicker in humans than in rodents. We studied epileptiform EEG spikes, bursts, and electrographic seizures; results might differ for behavioral seizures. The goal of this study was demonstration of a lasting effect of FUS on the EEG epileptiform activity. Ability of FUS to reduce numbers of electrographic seizures suggests a potentially useful clinical effect, but further investigations would be required to show actual lasting seizure reduction. Reduction of spiking was statistically significant, but modest in magnitude. Sham-injection animals (non-KA group) also presented some spikes and bursts, possibly due to surgical implant injuries or a baseline percentage of epileptiform activity in these rodent strains.

It is not possible to test all combinations of sonication parameters, including energy, frequency, pulse characteristics and duration, timing, and repetitions. Small changes in sonication parameters might have very different effects, in analogy to how small changes in stimulation frequency with repetitive transcranial magnetic stimulation can change inhibition to excitation [44]. The histological evaluation conducted in this study might not portray all adverse tissue effects, and this study did not evaluate potential cognitive effects of FUS. Astrocytes and microglia are associated with inflammation, but our study cannot identify the role inflammation plays in seizures or tissue injury. Lastly, we did not characterize the true long-term effects of FUS against spiking over months. Despite these limitations, this study suggests that certain regimens of FUS neuromodulation might provide sufficiently long-lasting benefits to be useful in the treatment of clinical epilepsy.

## Conclusion

Neuromodulatory pulsed-focused ultrasound can suppress EEG epileptiform discharges (spikes, bursts, and electrographic seizures) in a kainic acid model of epilepsy for up to 7 weeks. Higher-intensity regimens induce inflammation and tissue injury, but lower-intensity regimens delivered in one session with no apparent histological effects can still suppress spikes for up to 7 weeks. Although findings in laboratory models of epilepsy do not always translate to the clinical setting, the ability to demonstrate neuromodulatory changes lasting weeks provides hope that regimens can be developed with benefits sufficiently enduring to be useful for treating clinical epilepsy.

## Supplementary Information

Below is the link to the electronic supplementary material.

Supplementary file1 (DOCX 190 kb)

Supplementary file2 (DOCX 618 kb)

Supplementary file3 (PDF 433 kb)

Supplementary file4 (PDF 433 kb)

Supplementary file5 (PDF 439 kb)

Supplementary file6 (PDF 441 kb)

Supplementary file7 (PDF 434 kb)
