# Supplementary material for: Pulsed-Focused Ultrasound Provides Long-Term Suppression of Epileptiform Bursts in the Kainic Acid-Induced Epilepsy Rat Model
Source: Neurotherapeutics. 2022 May 17;19(4):1368–80. doi: 10.1007/s13311-022-01250-7 (PMC9587190; doi:10.1007/s13311-022-01250-7)
Supplement: Supplementary file 1 — Supplementary file1 (DOCX 190 kb) [file 13311_2022_1250_MOESM1_ESM.docx]

**SUPPLEMENTARY INFORMATION**


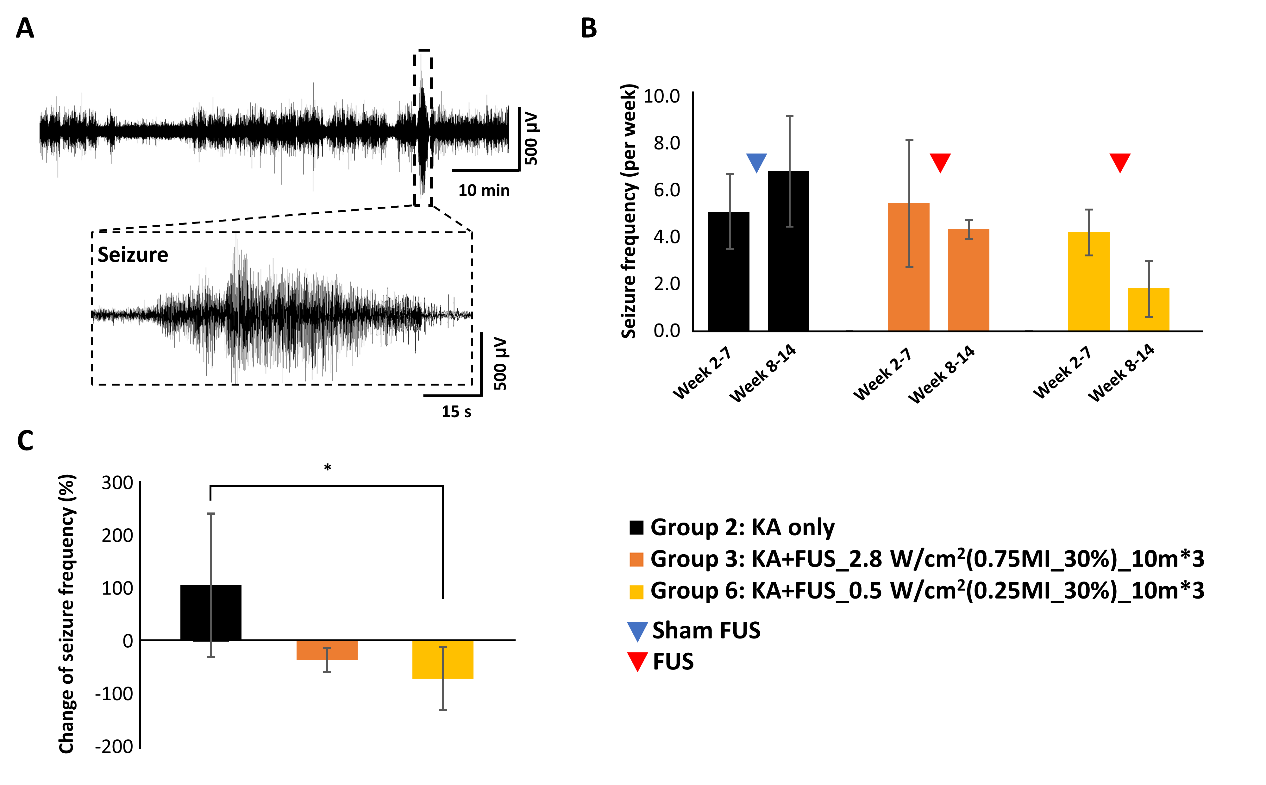


**Fig. S1.** Electrographic seizures with the KA model during the treatment period. (A) Typical example of the EEG signal and an electrographic seizure. (B) Seizure frequency of the KA animals under the sonication condition identical to group 3 (0.75-MI, 2.8 W/cm^2^, 30% duty cycle, 10 min. * 3) and group 6 (0.25-MI, 0.5 W/cm^2^, 30% duty cycle, 10 min. * 3), respectively. The triangles indicate sonication. (C) Change of seizure frequency (weeks 8-14 versus weeks 2-7) of the KA animals under the sonication condition identical to group 3 and group 6. *denotes a significant difference between groups, *p* < 0.05
