# Supplementary material for: Pulsed-Focused Ultrasound Provides Long-Term Suppression of Epileptiform Bursts in the Kainic Acid-Induced Epilepsy Rat Model
Source: Neurotherapeutics. 2022 May 17;19(4):1368–80. doi: 10.1007/s13311-022-01250-7 (PMC9587190; doi:10.1007/s13311-022-01250-7)
Supplement: Supplementary file 2 — Supplementary file2 (DOCX 618 kb) [file 13311_2022_1250_MOESM2_ESM.docx]

**SUPPLEMENTARY INFORMATION**


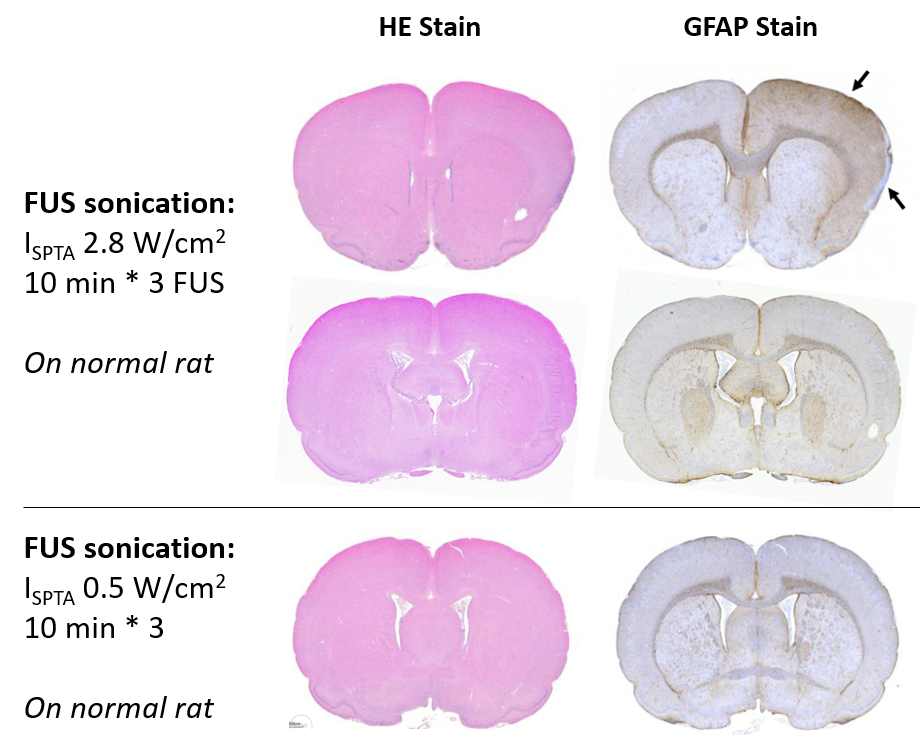


**Fig. S2.** Representative HE and GFAP staining of the normal animals under the sonication condition identical to group 3 (0.75-MI, 2.8 W/cm^2^, 30% duty cycle, 10 min. * 3) and group 6 (0.25-MI, 0.5 W/cm^2^, 30% duty cycle, 10 min. * 3), respectively. The black arrows indicate sites where inflammation responses induced by FUS.
